# Supplementary material for: Quantifying the impact of COVID-19 on immigration in receiving high-income countries
Source: PLoS One. 2023 Jan 19;18(1):e0280324. doi: 10.1371/journal.pone.0280324 (PMC9851539; doi:10.1371/journal.pone.0280324)
Supplement: S1 Appendix — (DOCX) [file pone.0280324.s001.docx]

Appendix I

**Sensitivity analysis**

To check the robustness of our models, we carried out a sensitivity analysis by using data for the 2012-2018 period to forecast the immigration count in 2019, rather than using data for the 2012-2019 period to predict the immigration count in 2020. We used the same methodology and ARIMA model specifications used to forecast 2020, except for Sweden and Switzerland. In these countries, root tests indicated that model specifications that best fitted the data were Random Walk with drift for the former and White Noise model for the latter. We do not expect observed and forecasted immigration counts in 2019 to differ as much as we do for 2020. We also expect observed immigration counts for 2019 to be within the 95% confidence intervals (CI) of the forecasted values for the same year. If observed and forecasted immigration counts for 2019 differed substantially and the former is outside the forecast CI, this would point to a flaw in our analytical approach.

Supplementary S1 Fig. reports observed immigration counts for 2012-2019 and forecasted values of 2019. Supplementary S2 Fig. reports the percentage difference between observed and forecasted immigration counts in 2019. Light circles indicate that observed values of 2019 are within the 95% confidence interval of forecasted immigration counts. The results of our sensitivity analysis show that observed immigration counts for 2019 are within the confidence interval of predicted immigration counts, except for Australia, where an unusual increase of immigration was observed. Differences between observed and forecasted immigration counts for 2019 are below 15% in all the counties, including Australia. Expected and observed immigration values are virtually the same for Spain, Norway, Canada and France. Differences are around 5% for the United States, Italy, Germany, Austria, Switzerland and Finland. The countries with the highest variations are Denmark, (9%), the Netherlands (11%), Ireland (12%), Sweden (13%) and Australia (14%).


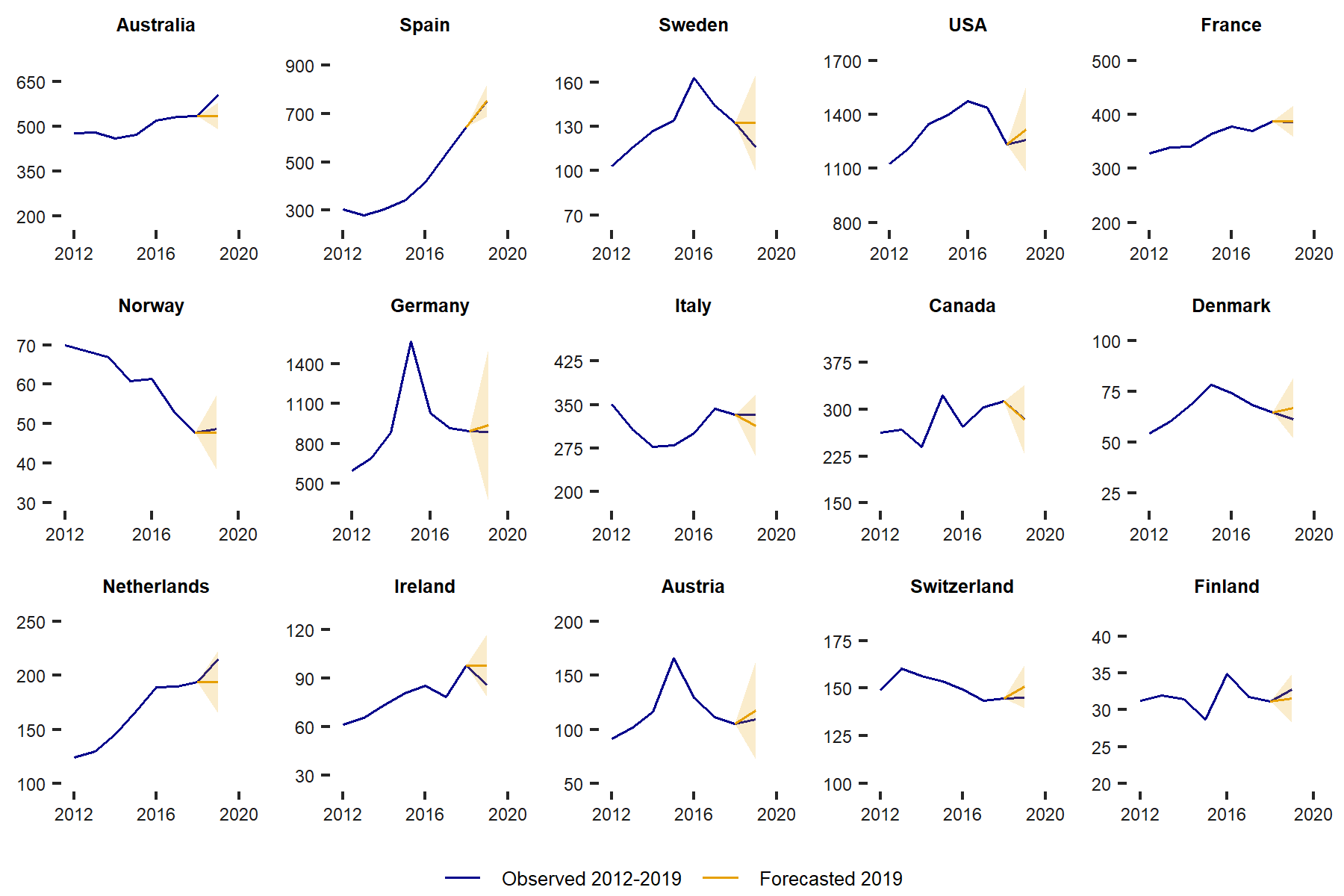


S1 Fig. Sensitivity analysis of immigration forecasting: observed data 2012-2019 and expected values in 2019 (thousands). Countries are ordered according to the extent of difference between the observed and forecasted immigration in 2020; 95% CIs are included with the forecast.


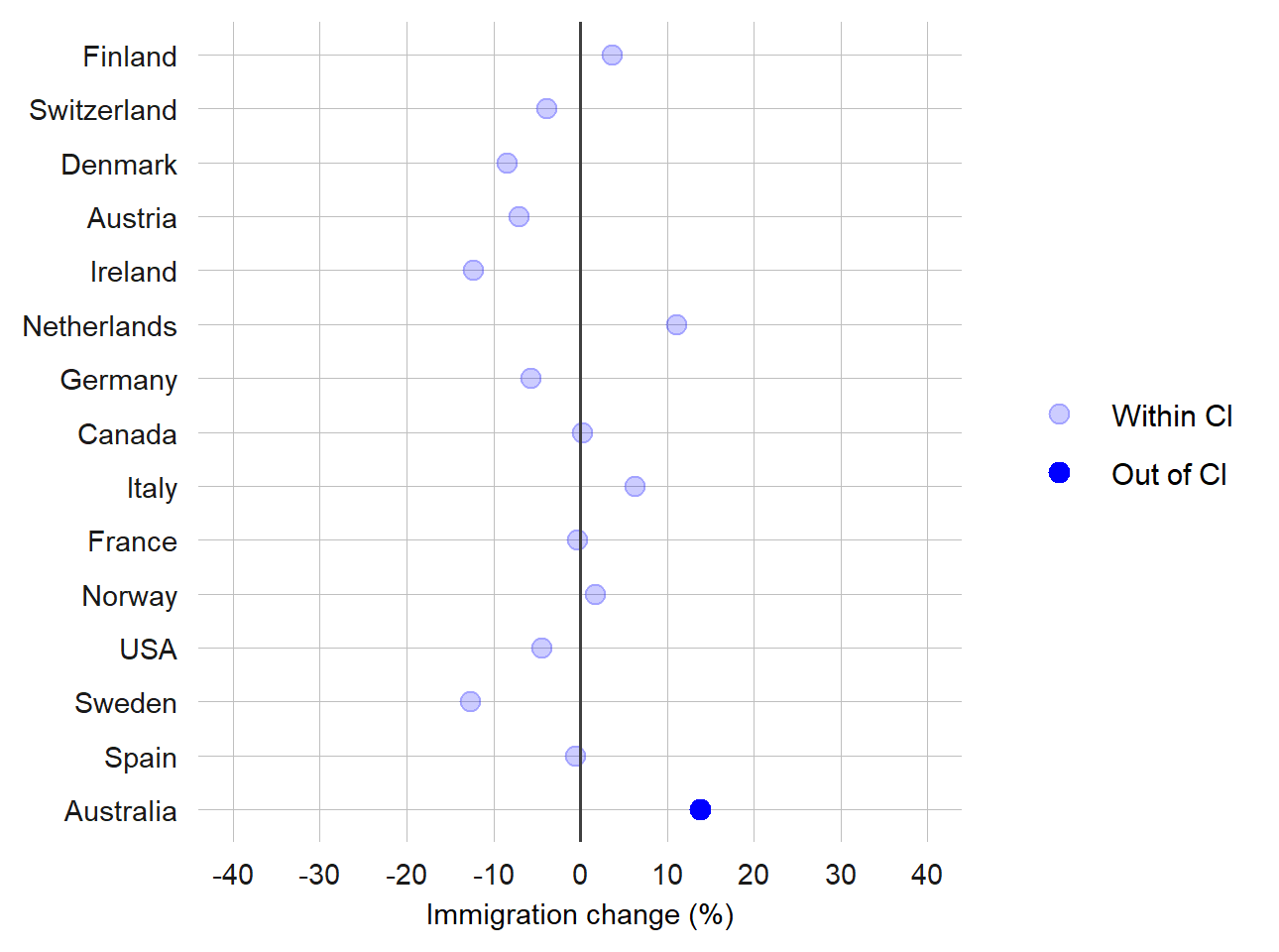


S2 Fig. Sensitivity analysis of immigration change between expected and observed values in 2019. Countries are ordered according to the extent of difference between the observed and forecasted immigration in 2020.
