# Supplementary material for: Quantifying the impact of COVID-19 on immigration in receiving high-income countries
Source: PLoS One. 2023 Jan 19;18(1):e0280324. doi: 10.1371/journal.pone.0280324 (PMC9851539; doi:10.1371/journal.pone.0280324)
Supplement: Attachment — Submitted filename: Peer Review Report; Manuscript Entitled - Quantifying the impact of COVID-19 on immigration in receiving high-income countries.docx [file pone.0280324.s002.docx]

***PEER REVIEW REPORT***

*Manuscript Number: PONE-D-22-22645*

*Entitled:*

***Quantifying the Impact of COVID-19 on Immigration in Receiving High-Income Countries***

*Author(s):* *Miguel González-Leonardo; Michaela Potančoková; Dilek Yildiz; Francisco Rowe*

***Review Criteria:***

1. **Methods:** Are the methods described in sufficient detail to understand the approach used and are appropriate statistical tests applied?

This paper empirically investigates the Impact of COVID-19 on Immigration in Receiving High-Income Countries. The main results stated in the current manuscript are 1) The Impact of COVID-19 on Immigration in Receiving High-Income Countries, and 2) The Impact of COVID-19 on Immigration in Receiving High-Income Countries by forecasting 35 counterfactual levels in 2020 assuming no pandemic and comparing these estimates with observed 36 immigration counts. Here are some comments and suggestions for the authors' consideration:

a) The contribution of the current manuscript is difficult to decipher, partially due to the 'Introduction' section not being well structured. It would be welcome if the authors could spend more time justifying the impact of covid-19 that underpins the research topic, previewing what the readers could expect to learn from the current study, and explaining how the authors' empirical investigation or research approach contributes to the existing literature. In any event, after the 'Introduction', one would expect to learn lessons and new information from this paper about the role and the mechanism of covid-19 in determining the impacts on immigration in receiving high income.
b) The empirical strategy adopted in the current manuscript could not serve the purpose. Using individual-level panel data, the current paper aims to estimate and quantify the Impact of COVID-19 on Immigration in Receiving High-Income Countries in each immigration destination on individuals' migration decisions. The authors approach this research question using a one-way (time) fixed effect logit regression model.
c) It could help if the authors could illustrate how the covid-19 is compared to other traditional factors (such as local job opportunities, education resources, social benefits, etc.) in terms of its predictive power on individuals' immigration decisions.
d) To facilitate discussions on the empirical results, it would help if more information on the data could be provided, e.g., a) how the covid-19 is constructed, what is its scale, how it should be interpreted, and summary statistics of the index by location and over time, and b) the decomposition and cross-tabulations of the migrants in terms of their education level, income, gender, and occupations.
e) It would be better if figures and tables were self-contained so the readers would have sufficient information for interpretation.
f) There are typos, as well as stylistic and grammatical problems, in the current manuscript. Therefore, the manuscript could benefit from thorough proofreading before submission.

Is that kind of research important for high-income countries?

If yes, please mention why and explain that with an example.

The paper is based on the various theoretical aspects of previous studies.

Throughout the manuscript most of the references are outdated.

This method is still insufficient and needs a really wide explanation.

The data sources are not clear and they should contain citations from previous studies, it will be more useful and clearer for the readers.

The data section should contain qualitative data and describe them well.

The authors did not describe the econometric methods as should as well in the study!

The econometric methods should be estimated correctly and must be revised to be clearer and to get all the necessary results of the study.

The model of the study is still not ordinarily modified and not briefly explained!

Which are the effects of ignoring the structural breaks in this analysis?

In the study, the authors used multi equations to analyze the data! Are these all equations necessary and should be used in this analysis? We need an explanation for that.

*The authors should consider the following studies, which are indexed in Scopus and Web of Science in their study such as:*

a) Aasen, A., N. Cavalli, L. Mencarini, S. Plach, and & S. Sanders. 2021. Early assessment of the relationship between the COVID-19 pandemic and births in high-income countries, Proceedings of the National Academy of Sciences of the United States of America 118(36): e2105709118. <https://doi.org/10.1073/pnas.2105709118>.
b) Aburto J.M., J. Schöley, I. Kashnitsky, L. Zhang, C. Rahal, T.I. Missov, M.C. Mills, J.B. Dowd, and R. Kashyap. 2021. Quantifying impacts of the COVID-19 pandemic through life expectancy losses: a population-level study of 29 countries, International Journal of Epidemiology 50(1): 63-74. https://doi.org/doi:10.1093/ije/dyab207.
c) IOM. 2020. COVID-19 Analytical Snapshot #3: Travel restrictions & mobility. Understanding the migration & mobility implications of COVID-19. Geneva: International Organization for Migration. https://www.iom.int/sites/default/files/our_work/ICP/MPR/COVID19_analytical_snapshot_3_-_travel_restrictions_and_mobility.pdf.
d) Newsham, N., and F. Rowe. 2019. Projecting the demographic impact of Syrian migration in a rapidly ageing society, Germany, Journal of Geographical Systems (23): 231-261. https://doi.org/10.1007/s10109-018-00290-y.
e) UN (2021). International Migration 2020 Highlights (ST/ESA/SER.A/452). Department of Economic and Social Affairs, Population Division, United Nations. https://www.un.org/development/desa/pd/sites/www.un.org.development.desa.pd/files/undes a_pd_2020_international_migration_highlights.pdf.
f) Wilson, C., Sobotka, T., Williamson, L., & Boyle, P. (2013). Migration and intergenerational replacement in Europe. Population and Development Review, 39(1), 131-157. https://doi.org/10.1111/j.1728-4457.2013.00576.x.
g) Wisniowski, A., Bijak, J., Christiansen, S., Forster, J. J., Keilman, N., Raymer, J., and Smith, P. W. (2013). Utilising expert opinion to improve the measurement of international migration in Europe. Journal of Official Statistics, 29(4):583–607. https://doi.org/10.2478/jos-2013-0041.
h) Hayward SE, Deal A, Cheng C, *et al* Clinical outcomes and risk factors for COVID-19 among migrant populations in high-income countries: a systematic review. J Migr Health 2021;**3**:100041.[doi:10.1016/j.jmh.2021.100041](http://dx.doi.org/10.1016/j.jmh.2021.100041)
i) Kondilis E, Papamichail D, McCann S. The impact of the COVID-19 pandemic on migrants, refugees and asylum seekers in Greece: a retrospective analysis of national surveillance data (Feb-Nov 2020): SSRN, 2021.
j) Marouf, F. E. (2017). Alternatives to immigration detention, Cardozo L. Rev. 38,101.
k) Sacchetti, M., and Hernández, A. R. (2020). ICE to stop most immigration enforcement inside the U.S. will focus on criminals during coronavirus outbreak, Washington, DC: Washington Post.

Please explain how could use the current model correctly by providing real examples.

Finally, the methods need to be modified and to be satisfied to reach the requested results of the study, because the methods are still weak somehow and should be revised in my opinion.

The methods need to be specific and chosen according to real and correct hypotheses.

Methodology and results generally look uncoherent.

Anyway, it is needed to better present results to give them the deserved meaning. In particular, I would suggest highlighting the connection of the results to the previous state hypotheses.

The baseline model consists of analyzing how public banks behave during and after a crisis.

The model needs more explanation and clarification.

The approach is not clear as should be. Please modify this issue well.

2. **Results:** Are the results or data that support any conclusions shown directly or otherwise publicly available according to the standards of the field?

Results: The research model is interesting! Although the research gap is not clear to me, can you provide a clear explanation?

The importance of work is not clear.

Please provide more descriptions to better understand readers.

The limitations are missing. The authors have to write the limitations of this study in sections 5 or 6.

The reviewed work's disadvantages should be mentioned in the related work, and a complete literature review should present mentioning the type (variety) of the study carried out.

Results and discussion: Given that this study deals with time-series data, the possibility of the non-stationarity of the variables cannot be ruled out.

There are some errors in the tables and can be changed the results and the conclusions.

The results section of panel regression still needs good clarification and must be improved.

The conclusion section still does not improve well according to the data analysis. Please develop it as should as be.

*However, the authors should consider some issues:*

- It appears that the paper is prepared in a rush.
- Mathematical modelling must be introduced to introduce to all readers all aspects, which characterize a regression analysis.
- The authors need to do more thorough work on the literature.
- The authors did not use a multiple linear regression model.
- The results should compare with other approaches, other than linear regression.
- This study could provide the opportunity and space for authors and researchers from various other countries to conduct other studies in the same field based on this study and its results.

*The authors should also consider the following:*

•	State the equation is missed and did not describe the method used.

•	The authors need to give a brief description of relevant theories.

•	The empirical part of this paper may need to be strengthened.

- English writing ability needs to be strengthened.
- I recommend the authors present the novelty of this research compared to previous research.
- It would be very useful to add in the "Introduction" section the purpose, objectives and hypothesis of the research.
- Language editing is mandatorily required to improve the quality of the paper.
- References should be written in a unified format.
- The authors must develop the conclusions.
- The literature section is missing in the study. This section should be written directly after the introduction section and should contain a large number of related previous studies.
- The state equation didn’t describe the method used and noticed that is still weak.
- The study variables used in descriptive statistics are unclear and must provide a statistical and logical description of the study problem.
- There is a need to add and improve the literature.

Thus, all macroeconomic data are expected to be subjected to a unit root test first to determine their stationarity. If data are found not to be stationary at all, then there is no need to regress them.

Unless otherwise stated, all regressing macroeconomic data that are not stationary can result in a spurious result, which can misguide policy direction.

What did the authors rely on in building and developing the conceptual framework for the study?

The authors must build this section in the body of the study. It will be more important and useful.

We need a clear explanation from which sources the authors have selected these data. Provide us with their sources.

The recommendations section after the concluding remarks is missing in the study. Where is it? Add this section please because it is very important in the study.

The authors should add this section at the end of the study after the conclusions section.

The policy implications are required and very essential for these types of studies.

The authors should add the declarations section correctly at the end of the study.

Recommendations for the results of this research need to be conveyed to the modelling after the authors added them.

It would be more useful to separate the conclusions section from the policy implications section in the study.

Which factors depended on the analysis of the study for the EU case on immigration in receiving high income?

Why do the authors rely on that? Provide us with an example and the main reasons for that.

The conclusion needs to be revised and developed. It is still weak somehow.

The empirical results of the study should be revised carefully again to fix all the errors in it.

I prefer to separate the empirical findings alone from the theoretical findings. That would be better and more useful for these types of studies.

These results in the empirical findings are considered primary results and need to be developed.

Explain that, please.

The results of the study are still weak and did not satisfy the methods, which were appropriate and used in the regression analysis, and did not satisfy the conclusions.

The results should be compared with conclusions carefully and should be clear to the readers.

The model did not use a suitable way to analyze the data, so the results are still weak and not satisfactory.

The conclusions must be based on the most important conclusions that can be drawn from the results.

The authors need to highlight the finding of the study in the abstract and the introduction sections.

The findings of the study have not been fully explained.

After the problems in the method are resolved, the new findings to be obtained should be rewritten by comparing them with the findings in the literature.

*My comments and suggestions for strengthening the are as follows:*

- I would recommend the author(s) clarify their research question.
- In the Introduction section, some paragraphs are too long and should be more condensed.
- Some terms of shallow integration and deep integration should be clarified and referenced.
- In addition, the authors might be considered to add one section on the current topic after the Literature review section.
- Conclusions could focus only on answering the research objectives, not summarizing the results or discussions.
- The policy implications must be mentioned and added at the end of the study. Where are they? Any policy implications could be put in a separate section.

It is strongly recommended to write a section on policy implications before conclusions.

On the other hand, I would suggest to the author(s) compare their results with the results of similar works.

The abstract is poorly written. It does not even include the methodology used in the paper.

Please clarify the abstract.

Provide an in-depth analysis of what and how not just generic narratives in the empirical results section.

Please provide the latest references up to the last 5 years.

Authors should discuss the limitations of their study and propose directions for future studies.

The introduction is not satisfactory enough. It is sometimes redundant.

I have relevant suggestions to make it more attractive and clearer to understand the relevance of your paper.

Clarify your proposal to fill the gap using specifically stated research questions or pointed objectives.

These would help the readers, even graphically, to better focus on your proposition.

3. **Interpretation:** Are the conclusions a reasonable extension of the results?

Please the authors clarify "subjective evaluation", or the author can provide a solid statistical theoretical basis that the item is not deleted because the purpose of your research is to build a model. If the instrument is not statistically valid, then your further-built model is not fit.

In my opinion, even though an instrument has a very established theoretical basis, in reality, the factor loading value did not prove, to meet the requirements, so it should be removed, because it could be, it will be the difference between your research and other research, right?

In the introduction to the study, the study needs to include many studies related to the topic and problem of the current study, and these studies must be included in Scopus, Web of Science.

The Interpretation of the study should receive more attention from the authors to be appropriate to the methods and findings of the study.

The authors should consider the interpretation more and it should be clearer.

In addition, the authors should return to the interpretation of the study and compare the results and methods with each other to reinforce the conclusion section and the recommendations of the study.

The interpretations must receive great, logical and systematic attention from the authors, and the interpretations must be consistent with the validity of the results and conclusions and be structured scientifically and logically according to the process of data analysis that took place during the study.

It would be more useful to develop the conclusion section of the policy implications section in the study.

The authors should arrange the declarations section correctly.

Do we need a clear explanation from which sources the authors have selected these data? Provide us with their sources.

The conclusions are a reasonable extension of the results, but there is a need for peer review in this study because of the conclusions extracted from the data analysis process.

The data must be analyzed more precisely and correctly than that, as there are errors and inaccurate numbers in the tables according to the sources that were used by the authors.

Therefore, the authors should carefully review the accounts and tables to reformulate the conclusions correctly and completely within the logic and according to the results of the study extracted after analyzing the available data.

Finally, we can say that the interpretation of the research results has been described in a weak method and should be taken again to describe one more time.

The authors should describe them by existing theories and relevant research.

The interpretation should be revised and developed strongly, correctly, and systematically.

Paragraphs should be made efficiently. The author simply explains the biographies of the respondents with the percentage only.

The interpretation is not sufficient as the authors need to test the effect of the proposed relationships and thus come out with meaningful conclusions.

I believe that the paper in its current form has fewer contributions. As the previous comment has not yet been addressed, the interpretation needs revisions as well.

Comments should be more satisfying and persuasive.

There are few citations in the sections after the literature part.

The findings of the study should be compared with the literature.

Models should be clearly explained.

Deficiencies in the study should be corrected by considering studies in reputable journals. The article should be revised considering how good work should be written.

The authors should write the motivation and contribution of the paper clearer.

The number of references is quite limited, should add more references about EU countries.

This research is heavily involved in the econometric application rather than using an appropriate economic theory and testing the hypotheses drawn from it.

Will be better to separate findings from the discussion and interpretation of the results.

There are some spelling mistakes across the text with very minor grammatical mistakes.

The authors also appear to use slangs, which should be carefully reviewed for consistency with the academic style of writing.

*My major comments on the manuscript are:*

- The introduction needs improvement. The authors should reorganize the structure of the introduction section to thoroughly express the aspects of this study including the background, current progress, motivation, the research question, objective, hypotheses, contribution, etc.
- The motivation and contribution need to be strengthened. The contribution should be expressed in a better way. In addition, expressions like firstly, secondly, and thirdly can be more useful in highlighting the contribution.
- The introduction and review sections seem to be overlapping in terms of references; the author should identify and eliminate them appropriately.
- The manuscript suffers from over and irrelevant referencing; the author tends to use too many references and especially secondary references throughout the manuscript which is a wrong practice.
- The reasonable and acceptable practice requires no more than 8 references in a sentence and they should be the ones that contributed to the existing literature in terms of theory, methodology or both.
- The literature review should be added and summarized with a summary table in chronological order, including author, period, country and result. This should be balanced in terms of author, country, and method so that there should not be an excess representation of any country, method, or authors.
- In the literature review section, you will critically examine previous literature and discuss the literature gap after the literature.
- The authors should refer to more recent papers and clarify the mechanisms of impact among the variables.
- The manuscript is a report with limited research hypotheses and a scientific explanation of the findings.
- The authors should present research hypotheses as well as the discussion of the empirical results. It needs to be extended and ameliorated to read more like a research paper.
- In the conclusion, please include a paragraph for limitations and future research directions.

4. **Ethics:** Do the study's design, data presentation, and citations comply with standard COPE ethical guidelines and have proper approval and consent been acquired as outlined in our Editorial Policies of PLOS ONE ethics?

In the current study, in short, the study design, presentation of data, and citations did not conform to COPE's standard ethical guidelines, and this matter should be thoroughly reviewed and revised, and adherence to COPE's standard ethical guidelines.

Authors must follow special editing policies according to PLOS ONE's ethics.

The authors in this study did not follow the required standard ethics, and the journal’s strict technical and ethical standards were not met.

Besides, there are missing parts in this section of the study.

Therefore, the authors must adhere to the technical and standard journal ethics, add the missing parts in the study and arrange them according to the principles of the journal's moral and guiding policy, principles to be followed.

*There are missing parts in the study (Ethics and Editorial Policies), such as:*

- Additional information
- Authorship (Author Contributions)
- Data Deposition
- Declarations section
- Raw Data
- Related Works
- Reporting Standards
- Statistical resources

***Comments to Authors: (Additional Evaluation)***

1. The manuscript requires a professional language revision (proofreading).
2. The authors must follow the publishing rules and the editorial policies of this journal well, and adjust the manuscript according to the rules of this journal and in an orderly manner.
3. The manuscript is within the scope of the journal.
4. It is preferable to support and strengthen the introduction better than that, and the focus must be on clarification more.
5. It is better to link references at the beginning of the paragraphs rather than at the end.
6. The hypotheses of the study are unclear.
7. The most important causes and factors that led to such incidents must be clarified as the possible means to properly control them.
8. The authors of this study can make good appropriate recommendations based on the findings of the study they reached.
9. The econometric model is unclear.
10. The Future Studies section must be added, it is important to have an explanation of major and important reasons for conducting future studies on this topic for the current study.
11. Researchers need to update the development of these theories.
12. The novelty of the study should be indicated in a special section at the end of the study after the conclusions section.
13. The theories of the study are still not clear and need more explanations.
14. The study was incorrect organized and needs to be organized well.
15. Language editing and proofreading are highly recommended for academic writing.
16. If the research problem and hypotheses can be stated clearly, it will be easy to understand the findings.
17. It is recommended to re-write the conclusion with more details while addressing the research problem and hypotheses while elaborating on the implications.
18. I suggest adding the study's significance for the government, policymakers and other regulatory bodies, the general public and other interested parties.
19. The justification of the study is missing.
20. The literature review needs up-gradation.
21. Some papers relating to PLOS ONE can be added.
22. Update references from 2018 to 2022.

**As it stands, this manuscript cannot be accepted for publication in the PLOS ONE journal until the authors complete all the required comments, suggestions and revisions.*
