# Supplementary material for: Quantifying the impact of COVID-19 on immigration in receiving high-income countries
Source: PLoS One. 2023 Jan 19;18(1):e0280324. doi: 10.1371/journal.pone.0280324 (PMC9851539; doi:10.1371/journal.pone.0280324)
Supplement: Attachment — Submitted filename: Responses_3.docx [file pone.0280324.s003.docx]

**Responses to reviewers for the manuscript #PONE-D-22-22645 “Quantifying the Impact of COVID-19 on Immigration in Receiving High-Income Countries”, submitted to PLOS ONE**

**REVIEWER #2**

Dear Authors,

*The topic of the paper is very interesting and very well presented. The analysis is very well conducted and the results are very interesting. However, there are some points I would like to point out.*

***1)*** *There is a lack of literature review, it would be interested to present excessively previous research on this topic which could provide more insights and explanations about your main aim. In addition, by providing literature review you can present more clearly the main scientific gap that you are trying to cover in this paper.*

Thank you for your constructive and supportive comments. Following the reviewer’s suggestion, we have conducted a new literature search and expanded the literature review in the introduction. We have found some studies on international migration during the pandemic, mainly focused on conceptual issues and stringency measures. Empirical studies remain limited and none of them analyses variations in international migration. Most contributions focus on how human mobility spread COVID-19 and how stringency measures increased involuntary immobility. To date, only one paper documented reductions on international migration in Spain during 2020 (González-Leonardo and Spijker 2022) and comparative empirical studies are entirely missing. We have included these papers and reinforced the gap of studies on how the pandemic affected levels of international migration across countries. See new text below:

“Human mobility and the spread of infectious diseases are closely related (Wesolowski et al. 2016). Several studies have demonstrated that human mobility patterns have influenced the ways in which COVID-19 has spread both across and within countries (e.g., Sirkeci and Yucesahin 2020; Chagas et al. 2021; Cheshmehzangi et al. 2021). National governments rapidly implemented mobility restrictions and border closures, among other stringency measures, to contain the spread of COVID-19 at the onset of the pandemic. Consequently, one of the major early impacts of the pandemic was the increase in involuntary immobility (Martin and Bergmann 2021). Both levels and patterns of international migration are likely to have been significantly affected: individuals with plans to migrate were not able leave their countries, others found themselves trapped in the migration journey or struggled to return to their home countries (Guadagno 2020, IOM, 2020).”

“Among the major receiving countries, immigration declines may have been more pronounced where there have been greater international travel restrictions (e.g., Australia and Canada) and other stringency measures, such as strict lockdowns and mobility restrictions (e.g., Italy). Additionally, it is expected that inflows to destinations traditionally receiving sizeable volumes of immigrants from large distances and overseas (e.g., Australia and Spain) have been more impacted, as travel restrictions mostly affected air travel (Guadagno, 2020). In sum, the closure of business and economic downturn caused by the pandemic decreased labor demand and increased unemployment in some countries (Blustein et al., 2020), constraining the potential need for international workers. Thus, country-specific variations in immigration levels are expected. To date, empirical studies estimating the impact of COVID-19 on international migration are surprisingly scarce. The extent to how much immigration declined during 2020 has been documented only in Spain, estimating a reduction of almost 40% (González-Leonardo and Spijker 2022). Therefore, cross-national variations on immigration due to COVID-19 are yet to be established.”

***2)*** *I am not sure why methods and materials are placed as a 4th part. I would suggest moving this part above the results. When writing a scientific paper it is better to have the methodology above the results as it makes it easier for the reader to understand the process you followed on your paper.*

We have restructured the paper as suggested. We have moved the method section before the results. Initially, we wanted to use a “research note” format, like that of PNAS, but we agree that the standard journal article structure is more appropriate.

***3)*** *On you apendix "supporting information" you present a lot of valuable information that should be placed inside the main text as it can complete parts such as methodology, results and conclusions.*

This is a fair point. We have moved text from the Supporting information (SI) section into the main text. All the text from the SI has been included in the methods section, except the Sensitivity analysis which is usually placed in the SI (appendix) in most empirical papers

***4)*** *It would be beneficial to add a part with limitations of your research, future research, and applications of your results.*

As suggested, we have included limitations, future lines of research and applications of our results in the discussion and conclusion section (we underlined the amended text)

“Results show reductions in immigration for most countries in our sample during 2020, except in Finland. Declines ranged from 5% in Switzerland to 60% in Australia but drops below 15% appeared not to be statistically significant due to the uncertainty levels of the forecast. Immigration trends are volatile and hard to predict due to complex and interrelated drives both in origin and destination (Bijak 2010; Hyndman & Athanasopoulos, 2018).”

“Differences in the content of stringency measures, rather than differences in their restrictiveness level, may explain the varying reductions in immigration. Australia, for instance, maintained strict restrictions to international travel from all countries during 2020, while the European Union (EU) and other states from the European Free Trade Association (EFTA) gradually relaxed travel restrictions for EU+EFTA citizens but maintained certain restrictions for other countries. Thus, EU+EFTA states which typically receive a large share of immigrants from the EU+EFTA territory (e.g., Finland) may have been less affected by immigration declines, while European countries with large proportions of immigrants from outside EU+EFTA (e.g., Spain) may have been more impacted. In addition, most inflows to Spain come by airplane from overseas, specifically from Latin America, and this mode of travel could have been more affected by border restriction. Otherwise, we only consider stringency measures and travel restrictions in receiving countries. Different stringency measures and pandemic impacts amongst sending countries, such as the lack of financial resources to travel due to the economic hardship caused by the pandemic, could have impacted levels of emigration at origin and, thus, immigration in destination countries as well.”

“As data for subsequent years become available, future work could expand our analysis to explore the prevalence and durability of declines in immigration across countries. Future research could also seek to understand the underlying factors explaining variations in the extent of immigration changes and how they come about by analysing the complexity of origin-destination migration flows. Analysis specific bilateral migration corridors can yield important insights and better interpretable results because some specific corridors may have been more impacted than others. However, such analysis would be possible only for a few countries due to limited data. Analysing origin-destination flows can enrich understanding of potentially transformative effects that COVID-19 may be having on the international migration system. Understanding these transformative effects is important given the fundamental role that migration plays in redistributing labour and skills to areas where they are needed, but also as a strategy to promote human development.”

In addition, we also amended text in the introduction to emphasise the utility of our result for policymakers (we underlined the amended text):

“International migration is the primary component of population change in aging societies. Net migration gains have been identified as a key demographic process to prevent or at least mitigate depopulation and labor force deficits (Lee, 2014; Abel 2018). Inflows of international migration increase the number of young adults and elevate fertility (Wilson et al., 2013; Newsham and Rowe 2021). They also bring labor force and skills where they are needed (Van Ham et al. 2001) and support the welfare state and intergenerational transfers by sustaining suitable labor dependency ratios (Lee et al 2014). Thus, monitoring movements between countries and understanding changes in the global network of international migration is essential to ensure appropriate policies in countries where natural change (difference between births and deaths) cannot sustain population growth and where labor force deficits exist.”

***5)*** *Please put a title on your figures and tables*

We have carefully revised the figures and tables. They all have a title. Could you please indicate where a title is needed? Thank you!

**REVIEWER #1. ASSISTANT PROFESSOR** **OF ECONOMICS AND FINANCE DR. NEMER BADWAN**

*All comments and suggestions on the current manuscript are available in this submission's attached peer review report. The Authors should take all comments and suggestions into consideration. This paper needs a major revision and modifications to be accepted for publication in PLOS ONE.*

***1. Methods:*** *Are the methods described in sufficient detail to understand the approach used and are appropriate statistical tests applied?*

*This paper empirically investigates the Impact of COVID-19 on Immigration in Receiving High-Income Countries. The main results stated in the current manuscript are 1) The Impact of COVID-19 on Immigration in Receiving High-Income Countries, and 2) The Impact of COVID-19 on Immigration in Receiving High-Income Countries by forecasting 35 counterfactual levels in 2020 assuming no pandemic and comparing these estimates with observed 36 immigration counts. Here are some comments and suggestions for the authors' consideration:*

Thank you for your comment. We are confused and do not fully understand the references to 35 counterfactual levels and 36 observed immigration counts. Our sample includes 15 countries, 8 years and we estimated counterfactual for one year.

a) *The contribution of the current manuscript is difficult to decipher, partially due to the 'Introduction' section not being well structured. It would be welcome if the authors could spend more time justifying the impact of covid-19 that underpins the research topic, previewing what the readers could expect to learn from the current study, and explaining how the authors' empirical investigation or research approach contributes to the existing literature. In any event, after the 'Introduction', one would expect to learn lessons and new information from this paper about the role and the mechanism of covid-19 in determining the impacts on immigration in receiving high income.*

We have carefully read the abstract and introduction and feel that the current text provides a clear justification for our analysis. We discussed the previous literature and identified an existing gap. The relevant text reads:

“Previous studies have examined the impact of COVID-19 on mortality and fertility. However, little is known about the effect of the pandemic on constraining international migration. We use Eurostat and national statistics data of immigration flows and ARIMA time-series models to quantify the impact of COVID-19 on immigration flows in 15 high-income countries by forecasting their counterfactual levels in 2020 assuming no pandemic and comparing these estimates with observed immigration counts. We then explore potential driving forces, such as stringency measures and changes in unemployment moderating the extent of immigration decline”.

Note: This comment is a bit puzzling. It is included in reference to methods, but it refers to the introduction.

b) *The empirical strategy adopted in the current manuscript could not serve the purpose. Using individual-level panel data, the current paper aims to estimate and quantify the Impact of COVID-19 on Immigration in Receiving High-Income Countries in each immigration destination on individuals' migration decisions. The authors approach this research question using a one-way (time) fixed effect logit regression model.*

We are afraid that there has been a misunderstanding of our paper. We do not use individual-level panel data. As stated in the methodology and data section, we use aggregate country-level data on immigration counts by year:

“We used annual official statistics on immigration flows from Eurostat online database (MIGR_IMM8) between 2012 and 2020 for the European countries, from the office of national statistics for Australia (Austrian Bureau of Statistics) and Canada (Statistics Canada), and from the Census Bureau for the United States. We restricted our analysis to immigration because of high levels of underreporting in emigration (Wisniowski et al., 2013). Immigrants are defined as individuals who lived over 1 year in the destination country, except for the United States, where immigration corresponds to the Census Bureau Vintage 2020 foreign-born immigration estimates”.

c) *It could help if the authors could illustrate how the covid-19 is compared to other traditional factors (such as local job opportunities, education resources, social benefits, etc.) in terms of its predictive power on individuals' immigration decisions.*

We struggle to see the relevance of this comment. The suggested analysis is outside the scope of our paper. It would require different data and methods. It seeks to address a different research question and the motivation of it is unclear to us.

d) *To facilitate discussions on the empirical results, it would help if more information on the data could be provided, e.g., a) how the covid-19 is constructed, what is its scale, how it should be interpreted, and summary statistics of the index by location and over time, and b) the decomposition and cross-tabulations of the migrants in terms of their education level, income, gender, and occupations.*

We are not entirely sure that we understand this comment. Assuming the Reviewer refers to what aspects of COVID-19 we considered in our analysis, we consider that changes in immigration during 2020 are most likely a result of COVID-19. Therefore, as explained in the methods section, we estimated a counterfactual in 2020 what would have been the levels of immigration if immigration trends were to continue as they were pre-pandemic, and we calculated the difference between the resulting estimate and the immigration count for the same year. The difference is deemed to reflect the impact of COVID-19.

Second, data attributes identified by the reviewer are not available. It is of common knowledge among migration scholars that such data do not exist. Some countries may have information only by sex and age, but it is not readily accessible or may be accessible with some lag.

e) *It would be better if figures and tables were self-contained so the readers would have sufficient information for interpretation.*

We are not sure what the reviewer means by “self-contained”.

f) *There are typos, as well as stylistic and grammatical problems, in the current manuscript. Therefore, the manuscript could benefit from thorough proofreading before submission.*

We have carefully revised and corrected typos throughout.

*Is that kind of research important for high-income countries? If yes, please mention why and explain that with an example.*

We already included discussion on why this study is important for high-income countries. For instance, see the second paragraph in the introduction and the third paragraph in the conclusion and discussion section.

Note: This comment is a bit puzzling. It is included in reference to methods, but it refers to other section.

*This method is still insufficient and needs a really wide explanation.*

This comment lacks any argument on what is wrong or indications of how to improve the method.

*The data sources are not clear and they should contain citations from previous studies, it will be more useful and clearer for the readers. The data section should contain qualitative data and describe them well.*

We are not entirely sure that we understand this comment. Which data from previous studies should we include? Which qualitative data is the Reviewer talking about? We explained the data sources we use. See response to comment d.

*The authors did not describe the econometric methods as should as well in the study!*

*The econometric methods should be estimated correctly and must be revised to be clearer and to get all the necessary results of the study.*

*The model of the study is still not ordinarily modified and not briefly explained!*

In the first version, we provided a detailed description of the modeling strategy in the Supplementary Information (SI). We have now integrated the SI into the methods section.

*Which are the effects of ignoring the structural breaks in this analysis?*

We are not entirely sure how this comment is relevant to our analysis. We do not conduct any time of structural break analysis.

*In the study, the authors used multi equations to analyze the data! Are these all equations necessary and should be used in this analysis? We need an explanation for that.*

We do not use multi equations. In any case, we could not find any entries to understand what multi equations are. It does not seem to be a recognizable method.

*The authors should consider the following studies, which are indexed in Scopus and Web of Science in their study such as:*

a) *Aasen, A., N. Cavalli, L. Mencarini, S. Plach, and & S. Sanders. 2021. Early assessment of the relationship between the COVID-19 pandemic and births in high-income countries, Proceedings of the National Academy of Sciences of the United States of America 118(36): e2105709118.* [*https://doi.org/10.1073/pnas.2105709118*](https://doi.org/10.1073/pnas.2105709118)*.*
b) *Aburto J.M., J. Schöley, I. Kashnitsky, L. Zhang, C. Rahal, T.I. Missov, M.C. Mills, J.B. Dowd, and R. Kashyap. 2021. Quantifying impacts of the COVID-19 pandemic through life expectancy losses: a population-level study of 29 countries, International Journal of Epidemiology 50(1): 63-74. https://doi.org/doi:10.1093/ije/dyab207.*
c) *IOM. 2020. COVID-19 Analytical Snapshot #3: Travel restrictions & mobility. Understanding the migration & mobility implications of COVID-19. Geneva: International Organization for Migration. https://www.iom.int/sites/default/files/our_work/ICP/MPR/COVID19_analytical_snapshot_3_-_travel_restrictions_and_mobility.pdf.*
d) *Newsham, N., and F. Rowe. 2019. Projecting the demographic impact of Syrian migration in a rapidly ageing society, Germany, Journal of Geographical Systems (23): 231-261. https://doi.org/10.1007/s10109-018-00290-y.*
e) *UN (2021). International Migration 2020 Highlights (ST/ESA/SER.A/452). Department of Economic and Social Affairs, Population Division, United Nations. https://www.un.org/development/desa/pd/sites/www.un.org.development.desa.pd/files/undes a_pd_2020_international_migration_highlights.pdf.*
f) *Wilson, C., Sobotka, T., Williamson, L., & Boyle, P. (2013). Migration and intergenerational replacement in Europe. Population and Development Review, 39(1), 131-157. https://doi.org/10.1111/j.1728-4457.2013.00576.x.*
g) *Wisniowski, A., Bijak, J., Christiansen, S., Forster, J. J., Keilman, N., Raymer, J., and Smith, P. W. (2013). Utilising expert opinion to improve the measurement of international migration in Europe. Journal of Official Statistics, 29(4):583–607. https://doi.org/10.2478/jos-2013-0041.*
h) *Hayward SE, Deal A, Cheng C, et al Clinical outcomes and risk factors for COVID-19 among migrant populations in high-income countries: a systematic review*. J Migr Health *2021*;***3***:*100041*.[*doi:10.1016/j.jmh.2021.100041*](http://dx.doi.org/10.1016/j.jmh.2021.100041)
i) *Kondilis E, Papamichail D, McCann S. The impact of the COVID-19 pandemic on migrants, refugees and asylum seekers in Greece: a retrospective analysis of national surveillance data (Feb-Nov 2020): SSRN, 2021.*
j) *Marouf, F. E. (2017). Alternatives to immigration detention, Cardozo L. Rev. 38,101.*
k) *Sacchetti, M., and Hernández, A. R. (2020). ICE to stop most immigration enforcement inside the U.S. will focus on criminals during coronavirus outbreak, Washington, DC: Washington Post.*

All above-listed studies are already referenced in our paper.

Note: This comment is a bit puzzling. It is included in reference to methods, but it refers to the literature review.

*Please explain how could use the current model correctly by providing real examples.*

We are not entirely sure that we understand this comment. What does the review mean by *real examples*?

*Finally, the methods need to be modified and to be satisfied to reach the requested results of the study, because the methods are still weak somehow and should be revised in my opinion.*

This comment lacks any argument on what is wrong and indications of how to improve the method.

*The methods need to be specific and chosen according to real and correct hypotheses.*

This comment has already been raised by the reviewer with different words. It lacks any argument on what is wrong and indications of how to improve the method.

*Methodology and results generally look uncoherent.*

It is unclear what the reviewer means. The comment lacks any argument on why the results are incoherent.

*Anyway, it is needed to better present results to give them the deserved meaning. In particular, I would suggest highlighting the connection of the results to the previous state hypotheses.*

*The baseline model consists of analyzing how public banks behave during and after a crisis.*

We struggle to see the relevance of this comment. We do not analyse how public banks behave.

*The model needs more explanation and clarification.*

This comment was already raised by the Reviewer. Please see previous responses.

*The approach is not clear as should be. Please modify this issue well.*

We are not able to address these general comments. They are unconstructive and lack any indication.

1. ***Results:*** *Are the results or data that support any conclusions shown directly or otherwise publicly available according to the standards of the field?*

*Results: The research model is interesting! Although the research gap is not clear to me, can you provide a clear explanation?*

*The importance of work is not clear.*

This comment was already raised by the Reviewer. Please see previous responses.

*Please provide more descriptions to better understand readers.*

We struggle to see the relevance of this comment. It lacks any indication on how to improve the manuscript.

The limitations are missing. The authors have to write the limitations of this study in sections 5 or 6.

The reviewed work's disadvantages should be mentioned in the related work, and a complete literature review should present mentioning the type (variety) of the study carried out.

Thank you for your comment. These issues have been addressed. See responses to comments 1 and 4 for Reviewer 2.

*Results and discussion: Given that this study deals with time-series data, the possibility of the non-stationarity of the variables cannot be ruled out.*

That is correct. We assessed and modelled for stationarity, seasonality and autocorrelation. Please see technical explanation in the methods section.

*There are some errors in the tables and can be changed the results and the conclusions.*

We have revised all the tables and have not found errors. Please provide information on where these errors are.

*The results section of panel regression still needs good clarification and must be improved.*

We do not use panel data analysis. We use ARIMA models.

*The conclusion section still does not improve well according to the data analysis. Please develop it as should as be.*

We struggle to see the relevance of this comment. It lacks any indication on how to improve the manuscript.

*However, the authors should consider some issues:*

- *It appears that the paper is prepared in a rush.*

This comment lacks any argument. The paper has been carefully prepared.

- *Mathematical modelling must be introduced to introduce to all readers all aspects, which characterize a regression analysis.*

Relevant equations have been included in the methods section.

- *The authors need to do more thorough work on the literature.*

This comment was already raised by the reviewer. Please see previous responses.

- *The authors did not use a multiple linear regression model.*

That is correct.

- *The results should compare with other approaches, other than linear regression.*

That is right. Other methods could be used to compare estimates with those produced in our paper.

- *This study could provide the opportunity and space for authors and researchers from various other countries to conduct other studies in the same field based on this study and its results.*

Thank you for your comment! We also believe that.

*The authors should also consider the following:*

*•	State the equation is missed and did not describe the method used.*

Relevant equations have been included in the methods section.

•	*The authors need to give a brief description of relevant theories.*

This comment was already raised by the reviewer. Please see previous responses.

Note: This comment is a bit puzzling. It is included in reference to results, but it refers to the method.

*•	The empirical part of this paper may need to be strengthened.*

This comment does not provide any indication of what should be addressed and how to do it.

- *English writing ability needs to be strengthened.*

This comment was already raised by the reviewer. Please see previous responses.

- *I recommend the authors present the novelty of this research compared to previous research.*

This comment was already raised by the reviewer. Please see previous responses.

- *It would be very useful to add in the "Introduction" section the purpose, objectives and hypothesis of the research.*

This comment was already raised by the reviewer. Please see previous responses.

- *Language editing is mandatorily required to improve the quality of the paper.*

This comment was already raised by the reviewer. Please see previous responses.

- *References should be written in a unified format.*

Thank you! The references have been checked.

- *The authors must develop the conclusions.*

We struggle to see the relevance of this comment. It does not provide any indication on how to develop the conclusion or what is lacking.

- *The literature section is missing in the study. This section should be written directly after the introduction section and should contain a large number of related previous studies.*

This comment was already raised by the reviewer. Please see previous responses.

- *The state equation didn’t describe the method used and noticed that is still weak.*

We are puzzled by this comment. The reviewer does not specify what component of the model is not clear and why.

- *The study variables used in descriptive statistics are unclear and must provide a statistical and logical description of the study problem.*

We have provided a description of the data and analysis. If more information is needed, it would be helpful if the reviewer could more clearly define what he considers is missing.

- *There is a need to add and improve the literature.*

This comment was already raised by the reviewer. Please see previous responses.

*Thus, all macroeconomic data are expected to be subjected to a unit root test first to determine their stationarity. If data are found not to be stationary at all, then there is no need to regress them.*

*Unless otherwise stated, all regressing macroeconomic data that are not stationary can result in a spurious result, which can misguide policy direction.*

Agree.

*What did the authors rely on in building and developing the conceptual framework for the study?*

*The authors must build this section in the body of the study. It will be more important and useful.*

This comment was already raised by the reviewer. Please see previous responses.

*We need a clear explanation from which sources the authors have selected these data. Provide us with their sources.*

This comment was already raised by the reviewer. Please see previous responses.

*The recommendations section after the concluding remarks is missing in the study. Where is it? Add this section please because it is very important in the study.*

*The authors should add this section at the end of the study after the conclusions section.*

*The policy implications are required and very essential for these types of studies.*

We have included some policy-oriented comments and future lines of research in the introduction and conclusion sections. See response to comment 4 for Reviewer 2.

*The authors should add the declarations section correctly at the end of the study.*

We are not allowed to include the declaration section in the manuscript in this stage.

*Recommendations for the results of this research need to be conveyed to the modelling after the authors added them.*

We are not entirely sure that we understand this comment.

*It would be more useful to separate the conclusions section from the policy implications section in the study.*

This is unusual in most journal articles.

*Which factors depended on the analysis of the study for the EU case on immigration in receiving high income?*

We are not entirely sure that we understand this comment.

*Why do the authors rely on that? Provide us with an example and the main reasons for that.*

We are not entirely sure that we understand this comment.

*The conclusion needs to be revised and developed. It is still weak somehow.*

This comment was already raised by the reviewer. Please see previous responses.

*The empirical results of the study should be revised carefully again to fix all the errors in it.*

We do not understand this unspecific comment. It lacks any indication on what is wrong, why and how to improve it.

*I prefer to separate the empirical findings alone from the theoretical findings. That would be better and more useful for these types of studies.*

We do not have theoretical results. It is an empirical paper.

*These results in the empirical findings are considered primary results and need to be developed.*

We struggle to see the relevance of this comment.

*The results of the study are still weak and did not satisfy the methods, which were appropriate and used in the regression analysis, and did not satisfy the conclusions.*

We are unsure which results the reviewer feels are weak and why. This comment lacks any argument.

*The results should be compared with conclusions carefully and should be clear to the readers.*

This comment was already raised by the reviewer. Please see previous responses.

*The model did not use a suitable way to analyze the data, so the results are still weak and not satisfactory.*

This comment was already raised by the reviewer. He did not provide any argument on why our results are weak.

*The conclusions must be based on the most important conclusions that can be drawn from the results.*

We struggle to see the relevance of this comment.

*The findings of the study have not been fully explained.*

We struggle to see the relevance of this comment.

*After the problems in the method are resolved, the new findings to be obtained should be rewritten by comparing them with the findings in the literature.*

The reviewer did not provide any argument on why our results are weak and why we should modify the method. As we already mentioned, there is no previous work on that topic to compare with.

*My comments and suggestions for strengthening the are as follows:*

- *I would recommend the author(s) clarify their research question.*

This comment was already raised by the reviewer. Please see previous responses

- *In the Introduction section, some paragraphs are too long and should be more condensed.*

In the introduction section, the longest paragraph has 8 lines.

- *Some terms of shallow integration and deep integration should be clarified and referenced.*

We are not clear what “shallow integration” / “deep integration” means. We do not use those terms in our article.

- *In addition, the authors might be considered to add one section on the current topic after the Literature review section.*

It is unclear what should be added.

- *Conclusions could focus only on answering the research objectives, not summarizing the results or discussions.*

We do not understand the direction of this comment. The objectives and research questions are answered from the results. Summarizing the main findings in the conclusions is a common practice.

- *The policy implications must be mentioned and added at the end of the study. Where are they? Any policy implications could be put in a separate section.*

This comment was already raised by the reviewer. Please see previous responses.

- *It is strongly recommended to write a section on policy implications before conclusions.*

This is an uncommon practice in journal articles. Policy implications are usually placed within the introduction and conclusion sections.

*On the other hand, I would suggest to the author(s) compare their results with the results of similar works.*

This comment was already raised by the reviewer. Please see previous responses.

*The abstract is poorly written. It does not even include the methodology used in the paper.*

*Please clarify the abstract.*

We have included the method we use in the abstract. This is the only issue we can address, as the comment lacks any other indication on how to improve the abstract. See new text underlined below:

“We use register data and ARIMA time-series models to quantify the impact of COVID-19 on immigration flows in 15 high-income countries by forecasting their counterfactual levels in 2020 assuming no pandemic and comparing these estimates with observed immigration counts”.

*Provide an in-depth analysis of what and how not just generic narratives in the empirical results section.*

We struggle to see the relevance of this comment. It lacks any indication of how to improve the narrative.

*Please provide the latest references up to the last 5 years.*

All the references, except two methodological papers, have been published in the last five years.

*Authors should discuss the limitations of their study and propose directions for future studies.*

This comment was already raised by the reviewer. Please see previous responses.

*The introduction is not satisfactory enough. It is sometimes redundant.*

We struggle to see the relevance of this comment. It lacks any argument on why the introduction is redundant and any direction on how to improve it.

*I have relevant suggestions to make it more attractive and clearer to understand the relevance of your paper.*

*Clarify your proposal to fill the gap using specifically stated research questions or pointed objectives.*

*These would help the readers, even graphically, to better focus on your proposition.*

We struggle to see the relevance of this comment. It lacks any indication on how to improve the manuscript. We clearly included the objectives and research question in the introduction.

2. ***Interpretation:*** *Are the conclusions a reasonable extension of the results?*

*Please the authors clarify "subjective evaluation", or the author can provide a solid statistical theoretical basis that the item is not deleted because the purpose of your research is to build a model. If the instrument is not statistically valid, then your further-built model is not fit.*

*In my opinion, even though an instrument has a very established theoretical basis, in reality, the factor loading value did not prove, to meet the requirements, so it should be removed, because it could be, it will be the difference between your research and other research, right?*

We are sorry, but we fail to understand what the above text means and how it is related to our study.

*In the introduction to the study, the study needs to include many studies related to the topic and problem of the current study, and these studies must be included in Scopus, Web of Science.*

This comment was already raised by the reviewer. Please see previous responses.

Note: This comment is a bit puzzling. It is included in reference to interpretation, but it refers to the literature review.

*The Interpretation of the study should receive more attention from the authors to be appropriate to the methods and findings of the study.*

We struggle to see the relevance of this comment. It lacks any indication of how to improve the paper.

*The authors should consider the interpretation more and it should be clearer.*

This comment says the same as the previous one, but with different words.

*In addition, the authors should return to the interpretation of the study and compare the results and methods with each other to reinforce the conclusion section and the recommendations of the study.*

We struggle to see the relevance of this comment. It lacks any indication on how to improve the manuscript.

*The interpretations must receive great, logical and systematic attention from the authors, and the interpretations must be consistent with the validity of the results and conclusions and be structured scientifically and logically according to the process of data analysis that took place during the study.*

This comment was already raised by the reviewer. It lacks any indication of how to improve the paper.

*It would be more useful to develop the conclusion section of the policy implications section in the study.*

This comment was already raised by the reviewer. Please see precious responses.

*The authors should arrange the declarations section correctly.*

This comment was already raised by the reviewer. We are not allowed to include this section in the manuscript in the stage.

*Do we need a clear explanation from which sources the authors have selected these data? Provide us with their sources.*

This comment was already raised by the reviewer. The data sources are listed in the abstract and introduction and explained in the method section in detail. Please see previous responses.

*The conclusions are a reasonable extension of the results, but there is a need for peer review in this study because of the conclusions extracted from the data analysis process.*

We struggle to see the relevance of this comment.

*The data must be analyzed more precisely and correctly than that, as there are errors and inaccurate numbers in the tables according to the sources that were used by the authors.*

*Therefore, the authors should carefully review the accounts and tables to reformulate the conclusions correctly and completely within the logic and according to the results of the study extracted after analyzing the available data.*

This comment was already raised by the reviewer. It lacks any argument, or any clear indication of what errors should be corrected.

*Finally, we can say that the interpretation of the research results has been described in a weak method and should be taken again to describe one more time.*

*The authors should describe them by existing theories and relevant research.*

*The interpretation should be revised and developed strongly, correctly, and systematically.*

This comment was already raised by the reviewer. Please see previous responses,

*Paragraphs should be made efficiently. The author simply explains the biographies of the respondents with the percentage only.*

We find our paragraphs in line with their common use. We did not work with a survey and, therefore, we do not have respondents in our data.

*The interpretation is not sufficient as the authors need to test the effect of the proposed relationships and thus come out with meaningful conclusions.*

We are not entirely sure that we understand this comment. It lacks any indication of how to improve the analysis.

*I believe that the paper in its current form has fewer contributions. As the previous comment has not yet been addressed, the interpretation needs revisions as well.*

The reviewer is unspecific in most of his comments and does not give any clear indications of what exactly should be addressed.

*Comments should be more satisfying and persuasive.*

We struggle to see the relevance of this comment. It lacks any indication of how to improve the narrative.

*There are few citations in the sections after the literature part.*

We struggle to see the relevance of this comment. It lacks any indication of where we should include references.

*The findings of the study should be compared with the literature*.

This comment was already raised by the reviewer. Please see previous responses.

*Models should be clearly explained.*

This comment was already raised by the reviewer. Please see previous responses.

Note: This comment is a bit puzzling. It is included in reference to interpretation, but it refers to the method.

*Deficiencies in the study should be corrected by considering studies in reputable journals.*

We struggle to see the relevance of this comment.

*The article should be revised considering how good work should be written.*

We struggle to see the relevance of this comment.

*The authors should write the motivation and contribution of the paper clearer.*

This comment was already raised by the reviewer. Please see previous responses.

*The number of references is quite limited, should add more references about EU countries.*

This comment was already raised by the reviewer. Please see previous responses.

*This research is heavily involved in the econometric application rather than using an appropriate economic theory and testing the hypotheses drawn from it.*

This seems to be a misunderstanding. We do not aim at any econometric application. We quantify reductions of immigration flows to selected countries during the COVID-19 pandemic in 2020.

*Will be better to separate findings from the discussion and interpretation of the results.*

We struggle to see the relevance of this comment.

*There are some spelling mistakes across the text with very minor grammatical mistakes.*

This comment was already raised by the reviewer. Please see previous responses.

*The authors also appear to use slangs, which should be carefully reviewed for consistency with the academic style of writing.*

We carefully revised the text, and we did not identify slags. Could the reviewer indicate where he thinks we use slang and suggest better wording?

*My major comments on the manuscript are:*

- *The introduction needs improvement. The authors should reorganize the structure of the introduction section to thoroughly express the aspects of this study including the background, current progress, motivation, the research question, objective, hypotheses, contribution, etc.*

This comment was already raised by the reviewer. Please see previous responses.

- *The motivation and contribution need to be strengthened. The contribution should be expressed in a better way. In addition, expressions like firstly, secondly, and thirdly can be more useful in highlighting the contribution.*

This comment was already raised by the reviewer. Please see previous responses. Concerning the last sentence, this, or other ways to express the same is a matter of writing style.

- *The introduction and review sections seem to be overlapping in terms of references; the author should identify and eliminate them appropriately.*

We are not entirely sure that we understand this comment. Could the reviewer indicate what exactly is overlapped?

*The manuscript suffers from over and irrelevant referencing; the author tends to use too many references and especially secondary references throughout the manuscript which is a wrong practice.*

We include the appropriate literature on the research topic. All references are published in high ranked journals. Could the reviewer indicate which references are irrelevant or secondary?

- *The reasonable and acceptable practice requires no more than 8 references in a sentence and they should be the ones that contributed to the existing literature in terms of theory, methodology or both.*

We do not use more than 5 references in any sentence. On the other hand, we are not aware of any rule on the number of references. Could the reviewer indicate where we can find this rule?

- *The literature review should be added and summarized with a summary table in chronological order, including author, period, country and result. This should be balanced in terms of author, country, and method so that there should not be an excess representation of any country, method, or authors.*

We struggle to see the relevance of this comment. This is not an art of the state on the topic, but an empirical paper. Could the reviewer provide any example of empirical paper published in high-ranked journal which include tables summarizing references?

- *In the literature review section, you will critically examine previous literature and discuss the literature gap after the literature.*

We are not entirely sure that we understand this comment.

- *The authors should refer to more recent papers and clarify the mechanisms of impact among the variables.*

The first comment was already raised by the reviewer. Please see previous responses. We are not entirely sure that we understand why the reviewer means by *mechanisms of impact among the variables.*

- *The manuscript is a report with limited research hypotheses and a scientific explanation of the findings.*

This comment was already raised by the reviewer. Our paper is a short empirical work with two research questions and descriptive findings.

- *The authors should present research hypotheses as well as the discussion of the empirical results. It needs to be extended and ameliorated to read more like a research paper.*

This comment says the same as the previous one.

- *In the conclusion, please include a paragraph for limitations and future research directions.*

This comment was already raised by the reviewer. Please see previous responses.

3. ***Ethics:*** *Do the study's design, data presentation, and citations comply with standard COPE ethical guidelines and have proper approval and consent been acquired as outlined in our Editorial Policies of PLOS ONE ethics?*

*In the current study, in short, the study design, presentation of data, and citations did not conform to COPE's standard ethical guidelines, and this matter should be thoroughly reviewed and revised, and adherence to COPE's standard ethical guidelines.*

*Authors must follow special editing policies according to PLOS ONE's ethics.*

*The authors in this study did not follow the required standard ethics, and the journal’s strict technical and ethical standards were not met.*

*Besides, there are missing parts in this section of the study.*

*Therefore, the authors must adhere to the technical and standard journal ethics, add the missing parts in the study and arrange them according to the principles of the journal's moral and guiding policy, principles to be followed.*

We follow standard COPE ethical guidelines. If the reviewer found any indication of violating, he should clearly mention where they are.

We have now adapted the initial manuscript to fit PLOS ONE style.

*There are missing parts in the study (Ethics and Editorial Policies), such as:*

- *Additional information*
- *Authorship (Author Contributions)*
- *Data Deposition*
- *Declarations section*
- *Raw Data*
- *Related Works*
- *Reporting Standards*
- *Statistical resources*

We are not allowed to include some of these items in the current manuscript (e.g., authorship, declaration).

We have made all data and code available in a GitHub repository. The link has been provided to the editor.

*Comments to Authors: (Additional Evaluation)*

1. *The manuscript requires a professional language revision (proofreading).*

This comment was already raised by the reviewer. Please see previous responses..

2. *The authors must follow the publishing rules and the editorial policies of this journal well, and adjust the manuscript according to the rules of this journal and in an orderly manner.*

This comment was already raised by the reviewer. Please see previous responses.

3. *The manuscript is within the scope of the journal*.

PLOS ONE is a multidisciplinary journal.

4. *It is preferable to support and strengthen the introduction better than that, and the focus must be on clarification more.*

This comment was already raised by the reviewer. Please see previous responses..

5. *It is better to link references at the beginning of the paragraphs rather than at the end.*

We struggle to see the relevance of this comment.

6. *The hypotheses of the study are unclear.*

This comment was already raised by the reviewer. Please see previous responses.

7. *The most important causes and factors that led to such incidents must be clarified as the possible means to properly control them.*

This comment was already raised by the reviewer. Please see previous responses.

8. *The authors of this study can make good appropriate recommendations based on the findings of the study they reached.*

This comment was already raised by the reviewer. Please see previous responses.

9. *The econometric model is unclear.*

This comment was already raised by the reviewer. Please see previous responses..

10. *The Future Studies section must be added, it is important to have an explanation of major and important reasons for conducting future studies on this topic for the current study.*

We guess that by *Future Studies section* the reviewer means possible follow-up research and open questions for future. This comment was already raised by the reviewer. Please see previous responses.

11. *Researchers need to update the development of these theories.*

This comment was already raised by the reviewer. Please see previous responses.

12. *The novelty of the study should be indicated in a special section at the end of the study after the conclusions section.*

We struggle to see the relevance of this comment. This is an uncommon practice. Could the reviewer cite any study in high-ranked journal in which this specific section exists after the conclusion?

13. *The theories of the study are still not clear and need more explanations.*

This comment was already raised by the reviewer. Please see previous responses.

14. *The study was incorrect organized and needs to be organized well.*

This comment was already raised by the reviewer. Please see previous responses.

15. *Language editing and proofreading are highly recommended for academic writing.*

This comment was already raised by the reviewer. Please see previous responses.

16. *If the research problem and hypotheses can be stated clearly, it will be easy to understand the findings.*

This comment was already raised by the reviewer. Please see previous responses.

17. *It is recommended to re-write the conclusion with more details while addressing the research problem and hypotheses while elaborating on the implications.*

This comment was already raised by the reviewer. Please see previous responses.

18. *I suggest adding the study's significance for the government, policymakers and other regulatory bodies, the general public and other interested parties.*

This comment was already raised by the reviewer. Please see previous responses

19. *The justification of the study is missing.*

This comment was already raised by the reviewer. Please see previous responses.

20. *The literature review needs up-gradation.*

This comment was already raised by the reviewer. Please see previous responses.

21. *Some papers relating to PLOS ONE can be added.*

We have included two papers published in PLOS ONE: Marois et al. 2020; Chagas et al. 2021.

22. *Update references from 2018 to 2022.*

This comment was already raised by the reviewer. Please see previous responses All our references were published in the last 5 years, except two methodological papers.

**As it stands, this manuscript cannot be accepted for publication in the PLOS ONE journal until the authors complete all the required comments, suggestions and revisions.*
